# The importance of a multidimensional approach to the preclinical study of major depressive disorder and apathy

**DOI:** 10.1042/ETLS20220004

**Published:** 2022-11-22

**Authors:** Megan G. Jackson, Emma S. J. Robinson

**Affiliations:** School of Physiology, Pharmacology and Neuroscience, University of Bristol, Bristol, U.K.

**Keywords:** animal model, apathy, behaviour, depression, negative valence

## Abstract

Both the neuropsychiatric syndrome of apathy and major depressive disorder comprise a heterogenous cluster of symptoms which span multiple behavioural domains. Despite this heterogeneity, there is a tendency in the preclinical literature to conclude a MDD or apathy-like phenotype from a single dimensional behavioural task used in isolation, which may lead to inaccurate phenotypic interpretation. This is significant, as apathy and major depressive disorder are clinically distinct with different underlying mechanisms and treatment approaches. At the clinical level, apathy and major depressive disorder can be dissociated in the negative valence (loss) domain of the Research Domain Criteria. Symptoms of MDD in the negative valence (loss) domain can include an exaggerated response to emotionally salient stimuli and low mood, while in contrast apathy is characterised by an emotionally blunted state. In this article, we highlight how using a single dimensional approach can limit psychiatric model interpretation. We discuss how integrating behavioural findings from both the positive and negative (loss) valence domains of the Research Domain Criteria can benefit interpretation of findings. We focus particularly on behaviours relating to the negative valence (loss) domain, which may be used to distinguish between apathy and major depressive disorder at the preclinical level. Finally, we consider how future approaches using home cage monitoring may offer a new opportunity to detect distinct behavioural profiles and benefit the overall translatability of findings.

## What do we mean by ‘apathy’?

Due to its multidimensional nature but current lack of known pathological mechanism, apathy is regarded as a neuropsychiatric syndrome [1–5]. As a syndrome, apathy is clinically and mechanistically distinct from major depressive disorder (MDD) and occurs alongside a range of neurodegenerative diseases [6, 7] as well as healthy aging [5]. It is defined as a primary loss of motivation that is not driven by emotional distress, intellectual impairment or diminished consciousness (e.g. drowsiness or delirium)[1]. The term ‘apathy’ discussed in the following review refers to the specific neuropsychiatric syndrome apathy. However, it should be acknowledged that apathy has also been described as a symptom of MDD, where it is used to describe a general loss of motivation and may be driven by other negatively valanced aspects of the disorder, such as low mood or anxiety. Here, we consider blunted emotional affect in combination with a reduction in reward motivation to be a hallmark of apathy syndrome behaviour, and distinguishes it from MDD at both the clinical and preclinical level.

Based on the early work of Levy and Dubois (2006) and more recently Robert and colleagues [8], apathy syndrome has been conceptualised into three domains of disrupted processing: behavioural-cognitive, emotional-affective and social motivation. Behaviours relating to the behavioural-cognitive domain include reduced motivation to start/finish a task. Behaviours relating to the emotional-affective domain include a lack of reaction to an external piece of news, be it positive or negative. Behaviours relating to the social motivation domain include a lack of initiation of social contact. To be diagnosed with clinical apathy, the patient must display behaviours relating to at least two of these domains, for a period of at least four weeks [8].

Both MDD and apathy consist of a cluster of heterogenous symptoms, yet many preclinical studies interpret MDD or apathy-related phenotypes based on a single behavioural measure. A valid animal model of neuropsychiatric disease should ideally not only recapitulate the mechanism by which it may occur, but also display a meaningful behavioural readout. Without this ethological validity [9], it is difficult to meaningfully conclude whether an animal model involves relevant underlying mechanisms and hence shows robust predictive validity [10]. The behavioural repertoire used to model MDD has expanded in recent years with several translational analogues now available [11–13]. However, there is a tendency in the preclinical literature to conclude a MDD phenotype based on the quantification of a single symptom of MDD, such as a deficit in motivation to work for reward, or a reduction in reward sensitivity, that may overlap with similar constructs such as apathy but arise from different underlying mechanisms. This is significant, as the neuropsychiatric syndrome apathy and MDD are clinically and mechanistically distinct [1, 14] and human imaging studies have found different underlying neurocircuitry and neuroanatomical changes in patient populations [15–17].

The development of the Research Domain Criteria (RDoC) [18] has re-evaluated the approach to defining neuropsychiatric symptoms taking into account their heterogenous constructs. MDD spans two domains within RDoC — the loss construct within the negative valence system and aspects of the positive valence system associated with deficits in reward processing [18]. Symptoms of apathy also span these two domains, however specific behaviours in the negative valence (loss) RDoC domain may distinguish apathy from MDD. In the context of MDD, negative valence (loss) has been characterised in both human studies and animal models of MDD and has been linked to disruptions which include but are not limited to: dysfunction in the fronto-cortical structures, disrupted reactivity to emotionally salient stimuli; changes to key genes related to monoamine neurotransmission; hypothalamic pituitary axis (HPA) dysfunction; and linked to an array of behaviours including negative bias in attention and memory, and low mood [18–21]. In contrast, apathy is characterised by an emotionally blunted state, with a reduction in behavioural and physiological response to emotionally salient stimuli [14, 22].

In this article, we focus on behavioural analysis as a tool in the assessment of preclinical models of MDD or apathy. We consider the importance of integrating behaviours across domains to strengthen model validity. We discuss how existing preclinical behavioural paradigms may map onto domains relevant to MDD and apathy, and consider how assessment of disruption to normal emotional response/affect within the negative valence (loss) system may be used to meaningfully dissociate apathy from MDD in animal models to the benefit of translational drug development.

## Differences in the underlying neurocircuitry of MDD and apathy

Symptoms of MDD span aspects of both the positive and negative valance (PV and NV) systems [18, 23]. PV describes responses relating to positive, rewarding experiences including reward learning and memory, reward sensitivity and motivation for reward [11, 24, 25]. These behaviours have been shown to engage brain regions including the frontostriatal pathways, lateral habenular, ventral tegmental area and nucleus accumbens [23, 26, 27]. Both rodent and human studies have shown that patients with MDD show impairments in these reward-related behaviours, with an associated reduction in activity in these brain regions compared with healthy controls [13, 28–30]. NV describes responses to unpleasant or aversive situations, which have been shown to engage brain regions such as the amygdala and anterior insula [23, 24, 31]. It has been found that some patients with MDD may show exaggerated emotional responses to aversive contexts or situations such as anxiety and fear. A meta-analysis of baseline activation and neural response has shown that patients with MDD have increased activity in these brain regions compared with healthy participants [32]. It has also been shown that patients with MDD show negative biases associated with emotional processing and cognition [12, 33, 34]. The combination of both exaggerated sensitivity to negatively valanced information and reduced or blunted processing of positive information have both been associated with changes in prefrontal and amygdala activity but may involve distinct neural networks within these regions [31, 35–37].

Changes in the neurocircuitry of those with apathy is less well characterised. However, a meta-analysis found that apathy is associated with disruptions in the fronto-subcortical circuitry [38]. Based largely on clinical observations from patients with brain lesions, it has been proposed that the distinct domains of apathy have differing underlying neurocircuitry. The emotional-affective domain is associated with the orbital/medial prefrontal cortex and limbic regions of the basal ganglia, while cognitive features of apathy are associated with dorsolateral prefrontal regions and cognitive areas of the basal ganglia such the global pallidus [14].

A number of studies have directly compared apathy and MDD in patient populations using resting-state fMRI and have shown distinct imaging profiles. In a population of Parkinson's Disease (PD) patients, it was shown that apathy score was best predicted by amplitude of the low frequency fluctuation (ALFF) in the right middle frontal cortex, left supplementary motor cortex and right orbitofrontal cortex. In contrast, MDD was predicted by ALFF in the right subgenual cingulate [15]. In another study, it was found that MDD in PD predicted increased functional connectivity between the orbitofrontal, hippocampal complex, caudate, thalamus and cingulate. In contrast, apathy predicted a decrease in functional connectivity between the orbitofrontal-parahippocampal circuit and caudate-thalamus circuit [16]. Therefore, both apathy and MDD consist of a cluster of symptoms relating to both the positive and negative valence domains but with divergence in terms of the profile of impairments and with evidence for distinct underlying neurocircuitry.

## How can apathy be distinguished from MDD at the clinical level?

Apathy and MDD share overlapping symptoms in the positive valence domain, including low motivation, psychomotor slowing, and diminished interest in previously enjoyed activities [5]. Comparison of commonly used apathy and MDD rating scales has shown that MDD and apathy can be most clearly dissociated in behaviours relating to the NV (loss) domain, specifically those of emotion/affect. Analysis of the Apathy Evaluation Scale (AES) and the Hamilton Rating Scale for Depression (HAMD) in 107 patients showed convergence in symptoms relating to impaired motivational state. When this section was removed, the correlation between scales disappeared [39]. This finding was consistent with another study, which compared the Cornell Scale for Depression (CSD) with the Frontal System Behaviour Scale (FrsBE) [6]. While apathy and MDD converged on symptoms relating to motivation, they diverged in symptoms relating to negative state and anxiety. Put simply, MDD in the negative valence domain can include periods of low mood and heightened negative emotional reactivity, while apathy is characterised by an emotionally blunted state. Therefore, while MDD and apathy may converge in the positive valence system, including deficits in motivation for reward, behaviours relating to the negative valence system including emotional reactivity may be utilised to distinguish a MDD phenotype from an apathy phenotype in animal models.

Apathy and anhedonia also show overlapping characteristics. Anhedonia is the inability to experience pleasure, and can also occur as a core symptom of MDD [26]. Anhedonia and apathy can be difficult to distinguish clinically, as a loss of pleasure in rewarding activities could be attributed to emotional blunting, characteristic of apathy. In line with this, apathy and anhedonia are highly correlated in some clinical populations. However, they do not fully overlap and can occur separately both clinically and in healthy populations [40, 41]. While anhedonia results in a blunted reward response, apathy results in a blunting of response to both positive and negative stimuli. Rodent studies also suggest that apathy and anhedonia have distinct neural substrates, and therefore may respond differently to treatment [42].

## The clinical importance of dissociating apathy syndrome from MDD

Apathy is one of the most common behavioural syndromes associated with neurodegenerative disease [43]. It appears early on in the disease and has a negative impact on disease progression. It has been associated with greater levels of functional disability and institutionalisation in Alzheimer's disease (AD) [44]. Apathy, but not MDD, has been shown to predict cognitive decline in AD [45] and has been shown to predict dementia in patients with cerebral small vessel disease [46]. Apathy is also a common behavioural syndrome associated with aging, occurring at a higher frequency than MDD [47]. In fact, one study showed that while the rate of apathy rose in healthy aged individuals over a period of 5 years, MDD declined [48]. Therefore, apathy represents an important early-stage behavioural syndrome distinct from MDD in aging and neurodegenerative disease which, if identified and targeted early on, may slow age or disease-related decline. It is currently unclear whether the behavioural domains seen in apathy domains are differently affected dependent on the primary pathology, but would represent an interesting avenue for future research. In further contrast with apathy, MDD may occur at a higher rate in other chronic conditions such as chronic pain. Pain and MDD are highly co-morbid and may each potentiate symptoms of the other, affecting disease duration and functional outcomes [49]. MDD is also associated poorer prognosis and more rapid disease progression in other chronic conditions such as ischemic heart disease and diabetes [50–52]. Conversely there is very little evidence of apathy syndrome in these chronic illnesses, though this may be down to a lack of research. Therefore, despite some overlapping symptoms, MDD and apathy occur at different rates in different health populations and life stages, and effective treatment may slow co-morbid disease progression. Effective treatment relies on distinguishing MDD from apathy syndrome, as the use of antidepressants in the treatment of apathy has little or no clinical evidence of efficacy (Benoit et al. 2008). A clinical trial studying remission rates in MDD found that worse severity of PV symptoms that overlap with apathy (low interest, lack of activity, lack of enjoyment) predicted poor outcomes to treatment with three different antidepressants [53]. Treatment of apathy with selective serotonin reuptake inhibitors (SSRIs), a first-line antidepressant, can exacerbate or induce apathy symptoms, known as SSRI-induced indifference [54].

## Limitations of a single dimensional approach in preclinical rodent models

The nature of apathy and MDD questionnaires allows for different symptom domains to be parsed relatively easily in patients. For example, by enquiring about the patient's motivation to start a task, how they would react to a positive or negative piece of news, or the last time they made effort to socialise, the interviewer gains information about the behavioural, emotional and social domains of apathy. However, these methods depend on subjective self-report measures which cannot be replicated in non-human species. As with many of the studies into psychiatric disorders, the use of subjective self-report measures is a major limitation to the development of translational approaches. One of the outcomes of the RDoC framework has been a shift in focus towards objective methods to define behavioural domains. This also provides an opportunity to develop translational methods for non-human species which build from the same underlying psychological constructs to generate more relevant behavioural tasks.

The operational definition of apathy gives an opportunity for the syndrome to be assessed as a quantifiable construct in animal models. However, reliance on the motivational deficit within this definition has led to the majority of preclinical studies approaching apathy as a unitary construct, with the focus on a quantifiable reduction in motivated behaviour. While the animal's willingness to physically work for reward is often probed, behaviours relating to the other two domains of apathy, such as its emotional state and social motivation are largely ignored. This makes it difficult to distinguish an apathy-like phenotype from a MDD-like phenotype in animals and potentially limits mechanistic insights. Reliance on a single domain or test may therefore result in either an apathy or MDD-like phenotype concluded from an animal model based on the chosen hypothesis. For example, studies have used the progressive ratio (PR) or effort-based decision making (EBDM) tasks in isolation to demonstrate apathy-related behaviour in rodent models of Huntington's Disease [55, 56]. Both of these tasks are well-validated measures of motivated behaviour with translational analogues [57, 58] and have been used to measure motivational state in many different pharmacological studies [59–61]. However, others have used the same tasks to model depressive symptoms and test the efficacy of antidepressants [62]. While motivation tasks do capture aspects of both apathy and MDD, it is not clear from one task alone which is being modelled and hence whether it engages relevant underlying mechanisms. This is important, as failure to engage clinically relevant targets at an early stage of the translational pipeline can have implications for the likelihood of success in terms of identifying clinically relevant drug targets. It is therefore important that we expand our approach at the preclinical level beyond a single behavioural test and utilise the opportunities presented by advances in objective methods being applied in clinical research.

## Apathy and MDD can be dissociated in the negative valence (loss) domain

**Figure 1.**
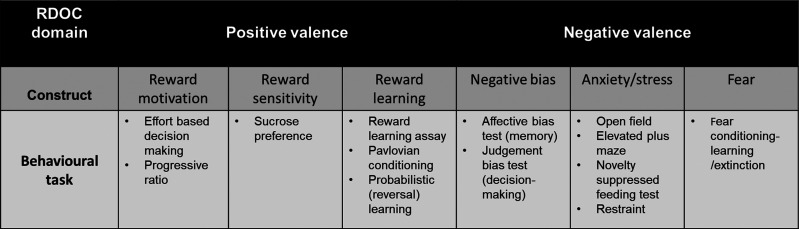
Examples of rodent behavioural tasks that map onto constructs within the positive and negative valence domains of the Research Domain Criteria relevant to both apathy and MDD. Apathy and MDD share overlapping symptoms in the positive valence domain such as impaired motivation for reward. However, they are dissociable in the negative valence domain. Symptoms of MDD may include negative affective state and an exaggerated response to emotionally salient stimuli. In contrast, apathy is characterised by a blunted response. Therefore, tasks such as the affective bias test, novelty suppressed feeding test and fear conditioning paradigms can be utilised alongside measures of reward processing to distinguish between apathy and MDD-related behaviour at the preclinical level.

Consistent with the clinical literature, rodent models of apathy and MDD should exhibit different types of impairments when considering the domain of emotion/affect within the NV (loss) system. Therefore, probing the response to emotionally salient stimuli in animal models, in addition to other behaviours, may facilitate the distinctions between apathy and MDD-like phenotypes at the preclinical level. This could potentially be achieved through the selection of translational analogues that map onto the relevant domains as illustrated in Figure 1.

Behaviours relevant to the NV domain in both apathy and MDD such as response to emotional stimuli and core affective state can be tested in rodents. Here, we focus primarily on rodent work, however it should be acknowledged that these behavioural domains can also be tested in higher species such as primates. Response to anxiety can be quantified using tasks based on rodent's natural aversion to open spaces and novelty, such as the novelty suppressed feeding test (NSFT). The NSFT has been used to test the efficacy of a range of anxiolytic treatments [63]. It has also been used in the study of various models of MDD, where increased levels of anxiety have been observed [64] and apathy where a blunted anxiety response was found [22]. Response to fear can be assessed using fear conditioning paradigms [65]. Fear conditioning has been used to test emotional blunting in schizophrenia patients. For example, a study found a reduction in aversion-related response and learning in patients with schizophrenia relative to healthy controls [66]. Similar findings were observed in an animal model of schizophrenia, where a reduction in freezing behaviour was found [67]. Conversely, patients with MDD were unable to inhibit their fear response in the presence of a safety cue, and rated stimuli as more emotionally arousing, leading to a reduction in discrimination learning compared with healthy controls [68]. Evidence for fear conditioning changes in MDD is mixed, with some finding an enhancement, and others no change [69, 70]. Animals with a depressive-like phenotype show enhanced fear response behaviours such as freezing as well as greater levels of anticipatory freezing [71]. Therefore if the negative valence (loss) domain is considered as a spectrum of disrupted emotional processing, apathy and MDD may be dissociated at the preclinical level in this domain.

The integration of these behaviours with additional units of analysis such as physiological response can benefit the overall interpretation of results. For example, changes in corticosterone, heart rate and skin conductance in response to emotional stimuli have been used as measures of both positive and negatively valanced emotional arousal [72–74] [75] and have been utilised in studies of both apathy and MDD [22, 76, 77]. As these measures can change in response to both positive and negatively valanced stimuli, it is important to localise physiological changes to specific behaviours.

As described above, MDD is also associated with negative affective biases in emotional processing and cognition [78]. Studies probing cognitive bias in tasks relating to emotional recognition, memory and categorisation have found that depressed patients show a negative bias in the interpretation and recall of information relative to healthy controls [36, 79]. Affective state can be investigated in rodents using the affective bias test (ABT), a bowl-digging task which assesses how affective state at time of reward learning impacts on reward memory in a subsequent choice test [80]. When rodents learn a cue-reward association under an induced negative affective state, they bias their choice away from that cue. Affective state can also be examined using the judgement bias test (JBT) [81]. This task tests how affective state affects reward-related decision-making under ambiguity. When rats are in an induced negative state, they are more likely to anticipate a less positive or a more negative event and are therefore more ‘pessimistic’ [82, 83].

**Figure 2.**
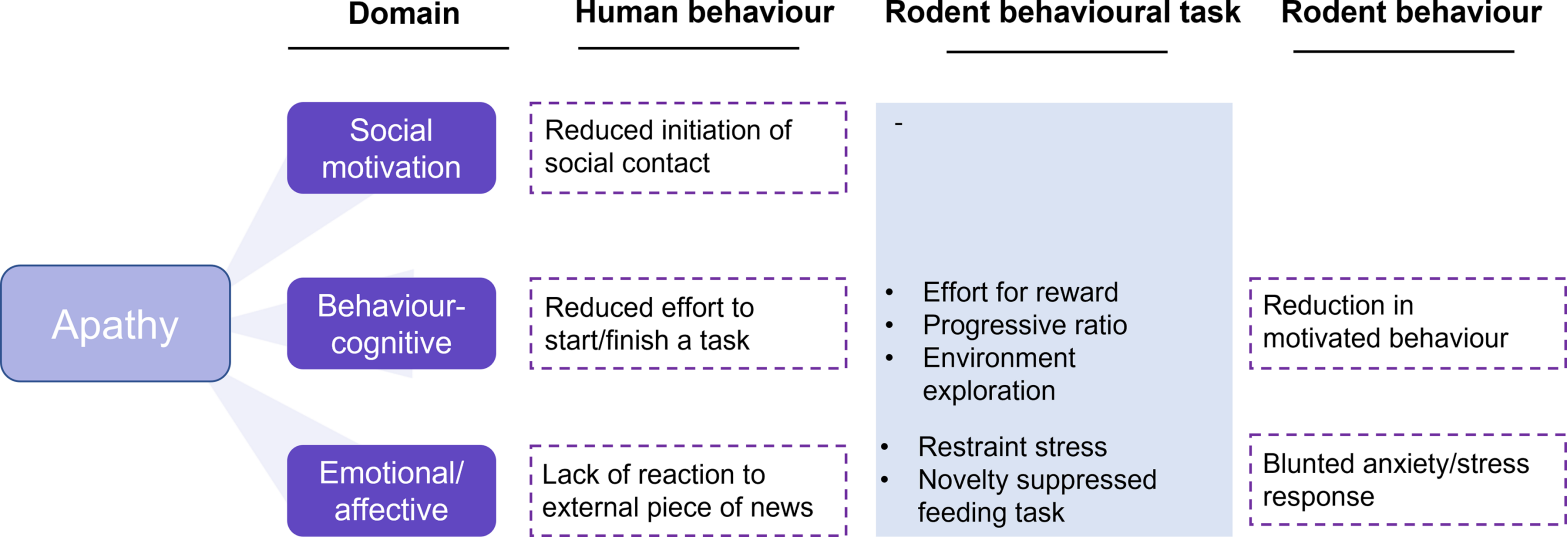
A multidimensional approach to the assessment of apathy-related behaviour in rodents utilised by [22]. The domains in dark purple represent the classification developed by [8]. Examples of human apathy behaviour relating to these domains are depicted alongside them. Rodent behavioural tasks hypothesised to map onto these domains and quantify these behaviours were used. These tasks spanned two domains of apathy and included behaviours relating to both the positive and negative valence domains of RDoC. From this, a behavioural profile distinct from what is usually found in MDD-like models was found.

Promisingly, there are some examples in the literature of a battery of behavioural tasks employed to parse MDD from apathy in phenotypic studies. Apathy is highly prevalent in Parkinson's Disease [7]. A study probing apathy-related behaviour in a genetic model of Parkinson's Disease [84] found a reduction in grooming/nesting behaviour, hypothesised to indicate apathy-related behaviour [85, 86]. This was observed alongside a reduction in reward sensitivity measured by the sucrose preference test (SPT), which is relevant to the emotional blunting domain of apathy. Measures of MDD-specific behaviour including low mood (forced swim test (FST)/tail suspension test (TST)) were used in combination with these other tasks to rule a MDD phenotype out. However, there has been some criticism about whether the FST/TST are meaningful measures of low mood [87]. Apathy is also common in otherwise healthy aging. In our study using aged male mice, behaviours in the behavioural-cognitive domain and the emotional-affective domain of apathy were tested (Figure 2). While rodent models of MDD typically show an increase in anxiety-related behaviour [85], aged mice showed a reduction in motivated behaviour alongside a blunting in anxiety behaviour and stress-reactivity, indicative of apathy rather than MDD-like phenotype [22]. Conversely, our study of a rodent model of MDD induced by early life adversity (ELA) showed that ELA rats were more sensitive to acute induction of a negative affective bias and showed a deficit in reward learning, independent of deficits in reward motivation and sensitivity [88].

**Figure 3.**
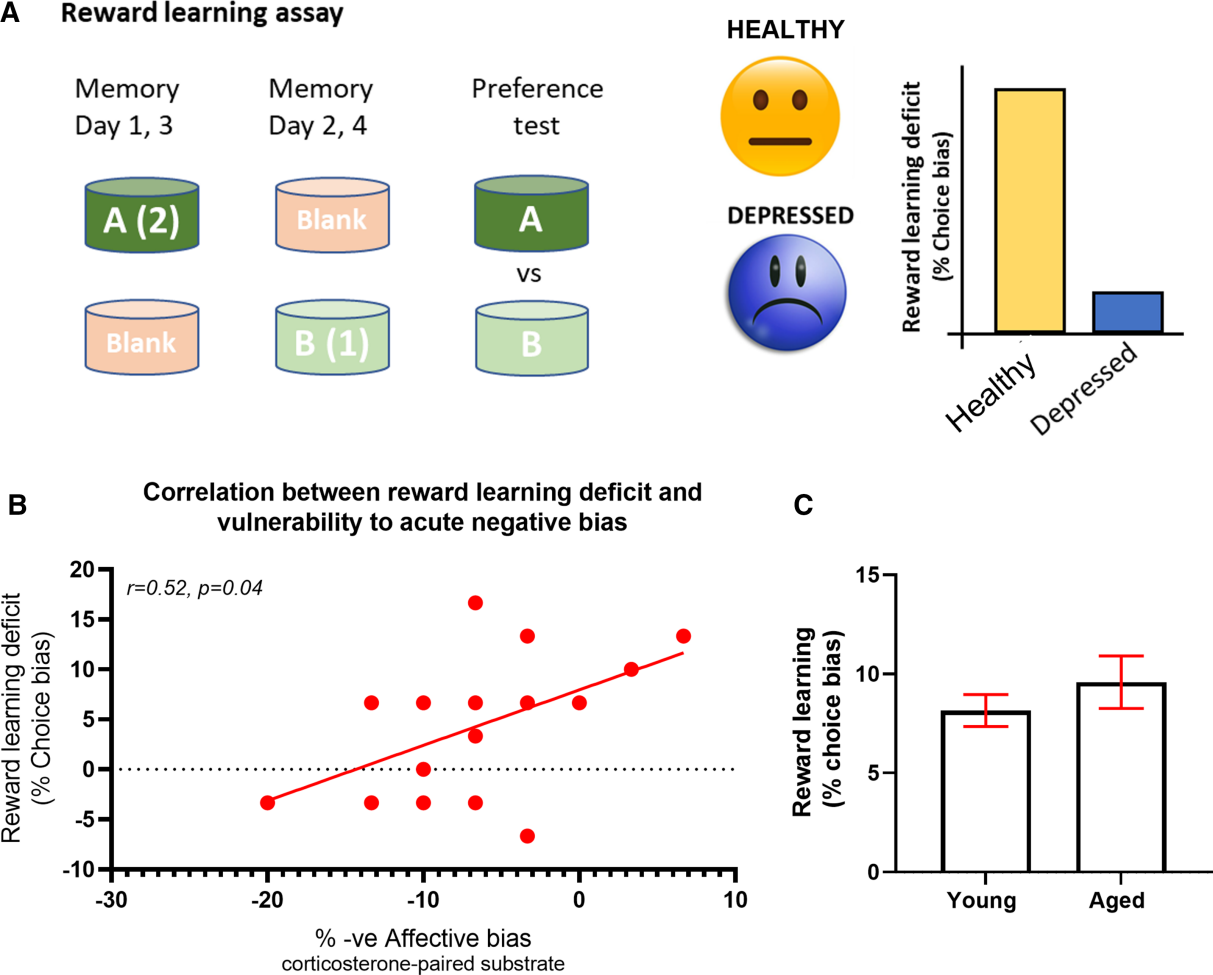
Vulnerability to acute negative bias is positively correlated with magnitude of a reward learning deficit induced by early life adversity. Reward learning was assessed using the reward learning assay depicted in (**A**). This assay consists of four learning sessions and a choice test. During the learning sessions the rodent is presented with two substrates. One is baited with either one or two reward pellets, and the other is a blank-it contains no reward pellets. This pairing is presented on days 1 and 3. On days 2 and 4 the rodent is presented with a different baited substrate containing the opposite number of pellets, and the same blank. In the choice test, the rodent is presented with the two previously baited substrates without the blank. Substrate choice over 30 trials is recorded and a % choice bias is calculated. In this way, reward learning and memory can be assessed. A normal rodent will show a positive bias towards the two-pellet paired substrate, while a MDD-like model will not show a bias. (**B**) Early life adversity rats with a greater magnitude of reward learning deficit in this assay were more vulnerable to acute negative bias induced by the administration of corticosterone as measured by the affective bias test. Data taken from [88]. (**C**) Healthy aging has been suggested as a model for apathy but aged rats show no impairment in reward learning, in contrast with the effects seen in MDD-like models [88, 89] (unpublished data, Jackson et al.).

It is also important to consider that behaviours or processes relating to one domain may impact behaviours in another. For example, using the reward learning assay (RLA) and ABT we have shown that vulnerability to acute negative bias is positively correlated with magnitude of a reward learning deficit induced by early life adversity (Figure 3). In addition to the deficits in this model, impairments in reward learning in the RLA have been observed in immune-mediated, corticosterone-induced and chronic pro-depressant drug-induced models of MDD [88, 89]. Impairments in reward learning also suggested a chronic negative affective state in a rodent model of chronic neuropathic pain [90]. Studies in a rat model of natural aging did not find impairments in reward learning (Figure 3) which suggests that this assay may differentiate between MDD and apathy phenotypes. However, these findings are preliminary and require further investigation in different apathy models. Taken together, this suggests that reward deficits relating to core affective state may also be used to distinguish between apathy and MDD at the preclinical level.

## Future perspectives

The use of discrete behavioural tasks can provide information on specific domains or components of behaviours. However, there is criticism that behavioural tasks are often very brief and highly sensitive to external conditions (light, time of day, handler experience, and so on). There are alternatives or methods that may be complementary to this approach, such as home cage monitoring. The use of home cage monitoring offers the opportunity to study a rodent's behaviour over extended periods, capturing a more comprehensive overview of their behavioural profile while minimising experimenter contact and potential for environmental confounds. It is also thought that this form of assessment is more reflective of response to pharmacological treatment in human clinical trials, which can last weeks-months [91, 92]. For example, the IntelliCage system has been utilised to assess a broad range of cognitive behaviours in a mouse model of AD over extended periods of time [93] and has been used assess the effects of environment and time on response to fluoxetine treatment in a chronic stress MDD-like model [94]. The assessment of waking inactivity has been shown to reflect a spontaneous MDD-like symptom [95]. The use of home cage monitoring may therefore be used to detect subtle changes and distinct behavioural profiles which may help distinguish between similar psychiatric syndromes in the future. This approach is further enhanced by the opportunities afforded by advances in computational and machine learning methods which can be used to automate the analysis of these large data-sets [91, 96].

## Conclusion

To create meaningful preclinical models for psychiatric drug development it is imperative that there is a shift away from single dimension behavioural tasks used in isolation. Apathy and MDD share some overlapping symptoms in the PV domain which makes them difficult to distinguish at the preclinical level. By considering the NV (loss) domain as a spectrum of disrupted processing whereby responses can be exaggerated or blunted, apathy and MDD phenotypes may be distinguished. This can be achieved by using a battery of behavioural tests that span behaviours within both the NV and PV domains, or potentially, the use of a complex behavioural analyses within enriched environments that measure aspects of both the NV and PV domains. Together, this will ensure the development of preclinical models of apathy and MDD with robust ethological validity and increase the likelihood of translating findings in laboratory animals to the clinic.

## Summary

- Both major depressive disorder (MDD) and the syndrome of apathy consist of a heterogenous range of symptoms, and more meaningful interpretation of rodent models could be achieved if behavioural phenotyping and integration of findings across domains is employed.
- Apathy and MDD share overlapping symptoms in the positive valence domain such as a reduction in motivation for reward, but may be differentiated in the negative valence (loss) domain of RDoC.
- Automated home cage monitoring could provide new opportunities to detect distinct behavioural profiles and ultimately help distinguish between similar psychiatric syndromes.

## Open Access

Open access for this article was enabled by the participation of University of Bristol in an all-inclusive *Read & Publish* agreement with Portland Press and the Biochemical Society under a transformative agreement with JISC.

## Abbreviations

ABT: affective bias test
AD: Alzheimer's disease
AES: apathy evaluation scale
CSD: Cornell scale for depression
EBDM: effort based decision making
EfR: effort for reward
ELA: early life adversity
FrsBE: frontal system behaviour scale
FST: forced swim test
HAMD: Hamilton rating scale for depression
JBT: judgement bias test
MDD: major depressive disorder
NSFT: novelty supressed feeding test
NV: negative valence
PD: Parkinson's disease
PR: progressive ratio
PV: positive valence
RDoC: research domain criteria
RLA: reward learning assay
SPT: sucrose preference test
TST: tail suspension test
